# Incorporating Lower Urinary Tract Symptoms to Predict the Risk of Positive Prostate Magnetic Resonance Imaging

**DOI:** 10.1016/j.euros.2026.05.014

**Published:** 2026-06-13

**Authors:** Leonor Jane Paulino Pereira, Harm H.E. van Melick, Michiel J.P.M. Sedelaar, Marco H. Blanker, Pim J. van Leeuwen, Roderick C.N. van den Bergh

**Affiliations:** a Department of Urology, St. Antonius Hospital, Nieuwegein/Utrecht, The Netherlands; b Department of Urology, Radboud University Medical Center, Nijmegen, The Netherlands; c Department of Primary and Long-Term Care, University Medical Center Groningen, Groningen, The Netherlands; d Department of Urology, Antoni van Leeuwenhoek Hospital, The Netherlands Cancer Institute, Amsterdam, The Netherlands; e Department of Urology, Erasmus University Medical Center, Rotterdam, The Netherlands

**Keywords:** IPSS, Lower urinary tract symptoms, Magnetic resonance imaging, Prostate cancer

## Abstract

**Background and objective:**

Many men referred from primary care entering magnetic resonance imaging (MRI)-based prostate cancer (PCa) pathways have an MRI without abnormalities, highlighting the need to improve MRI risk stratification. Optimising this process in primary care could reduce unnecessary referrals and MRIs. This study aimed to develop a prediction model incorporating the International Prostate Symptom Score (IPSS) to improve MRI risk stratification and reduce hospital referrals and MRIs in biopsy-naïve men with suspected PCa in primary care.

**Methods:**

We prospectively identified men with suspected PCa referred from primary care to a Dutch teaching hospital in 2022–2023. Standard work-up included IPSS and upfront biparametric MRI. Study outcomes included Prostate Imaging Reporting and Data System (PI-RADS) ≥ 4, the number of potentially reduced hospital referrals and subsequent MRIs, and missed PCa cases. Men with an abnormal digital rectal examination (DRE) were excluded from model development, as they have a direct indication for MRI according to current guidelines. Multivariable logistic regression identified predictors of PI-RADS ≥ 4. Model performance was assessed using the area under the curve (AUC), and clinical utility was evaluated with decision curve analysis using a predefined threshold probability of 20%.

**Key findings and limitations:**

Of 409 men, 334 without abnormal DRE were included in the model development cohort; 30% (101/334) had PI-RADS ≥ 4, of whom 59% (60/101) had significant PCa (International Society of Urological Pathology [ISUP] grade group ≥ 2). Prostate-specific antigen (PSA) and IPSS were independent predictors of PI-RADS ≥ 4. The model showed fair discrimination (AUC = 0.68; 95% confidence interval [CI] = 0.62–0.74). Decision curve analysis showed greater net benefit than ‘treat none’ across all thresholds and greater net benefit than ‘treat all’ between 17% and 30%. At a 20% threshold, 23% of MRIs could be avoided, while 3.9% of all patients would not be referred despite having a positive MRI (13% of positive MRI findings), including eight cases of missed significant PCa. Limitations include the lack of external validation.

**Conclusions and clinical implications:**

Incorporating IPSS in primary care for biopsy-naïve patients with suspected PCa improves risk stratification for MRI and offers an easily available parameter to optimise diagnostic pathways.

## Take Home Message

We developed a model incorporating lower urinary tract symptoms severity to improve magnetic resonance imaging (MRI) stratification in biopsy-naïve men with suspected prostate cancer in primary care. This approach could potentially reduce unnecessary hospital referrals and MRI scans by 23% while maintaining high detection rates of clinically significant prostate cancer.

ADVANCING PRACTICE **What does this study add?**

This is the first study to develop a model incorporating lower urinary tract symptoms (via the IPSS questionnaire) to improve MRI risk stratification for biopsy-naïve men with suspected PCa in primary care. By incorporating the IPSS, the model reduced unnecessary hospital referrals and MRIs by 23% while maintaining high clinically significant PCa detection rates. This approach offers an efficient, cost-effective strategy to optimise resource use and improve patient care.

**Clinical Relevance**

This article explores the effect of urinary symptoms, quantified with the help of the IPSS questionnaire, on the indication of doing MRI for prostate cancer diagnosis. The data shows that higher IPSS scores are protective to find a positive MRI (PIRADS >4). This might be used to better select patients for MRI scanning and with this reduce the amount of unnecessary MRIs an associated costs and resources. External validation is lacking. Editor-in-Chief: Jochen Walz, MD.

**Patient Summary**

This study addressed whether the severity of urinary symptoms could help general practitioners decide which men with suspected prostate cancer need referral and an MRI. We found that men with more severe urinary symptoms are more likely to have normal MRI results. By considering urinary symptoms alongside prostate-specific antigen levels, unnecessary referrals and MRI scans can be avoided without missing many cancers.

## 1 Introduction

An optimal prostate cancer (PCa) detection pathway should effectively identify high rates of clinically significant PCa (csPCa) and minimise the detection of clinically nonsignificant PCa (cnsPCa), while minimising the diagnostic burden. In biopsy-naïve men with elevated prostate-specific antigen (PSA) levels and/or abnormal digital rectal examination (DRE), prebiopsy magnetic resonance imaging (MRI) pathways have been shown to outperform systematic biopsy-guided pathways in csPCa detection, reducing overdiagnosis and biopsy indications [1], [2], [3]. In addition to effective risk assessment for biopsy, MRI also allows for targeted biopsy procedures and disease staging [4]. However, up to 55% of MRIs performed in patients with elevated PSA levels and/or abnormal DRE result in Prostate Imaging Reporting and Data System (PI-RADS) <4 [3], [5], [6], [7], [8]. Despite MRI’s high negative predictive value of 86–94%, which provides reassurance to men with negative results, many still undergo unnecessary MRI scans [9].

Few studies have addressed pre-MRI stratification tools to reduce unnecessary MRIs. Mannaerts et al [1] suggested that the Rotterdam Prostate Cancer Risk Calculator (RPCRC) could avoid 37% of MRIs in biopsy-naïve menKlik of tik om tekst in te voeren.. Similarly, the Prostate Health Index test and the Stockholm3 blood test could reduce MRIs and biopsies by 38% and 25%, respectively [10], [11]. However, these methods require additional transrectal ultrasound (TRUS) procedures (to calculate PSA density) or biomarker testing, leading to increased hospital visits, burden and costs [1], [10], [11]. Development of a simplified stratification method to identify who should undergo MRI may help to mitigate these associated challenges.

Elevated PSA levels in men with negative MRI results may be caused by other factors such as benign prostatic hyperplasia (BPH) [4], [12]. BPH involves cell proliferation in the transitional zone of the prostate, which may result in lower urinary tract symptoms (LUTS) [12]. Currently, LUTS has not been incorporated into multivariable PCa risk stratification tools, despite its inclusion in early risk calculators such as the European Randomized Study of Screening for PCa (ERSPC) [13]. Importantly, the prevalence of LUTS may be higher in general practitioner (GP)-referred cohorts when compared with screening cohorts, potentially enhancing their discriminatory value for identifying patients at risk of PCa.

Therefore, the aim of this study was to develop a prediction model incorporating LUTS, measured by the International Prostate Symptom Score (IPSS), to optimise MRI stratification and reduce hospital referrals and MRIs in biopsy-naïve patients with suspected PCa in primary care.

## 2 Material (patients) and methods

This cohort study was conducted at St Antonius Hospital in Utrecht, the largest nonacademic hospital in the Netherlands, from September 2022 to September 2023. Eligible patients, identified prospectively, were adult men referred by their GP with PSA levels of 3–50 ng/ml and/or DRE ≥ T2. Standard diagnostic work-up included the IPSS questionnaire and biparametric prostate MRI (bpMRI). Exclusion criteria included contraindications to MRI, use of 5-alpha-reductase inhibitors, or previous prostate MRI, biopsy, or PCa diagnosis. The study was approved by the Medical Research Ethics Committees United (W23.076).

All patients were referred by their GP, who assessed patient characteristics, PSA levels and DRE. Upon referral to the urology department, patients were sent the IPSS questionnaire digitally for completion prior to their first hospital visit. Subsequently, all patients underwent an upfront bpMRI.

The MRI protocol used a 3-Tesla scanner with a pelvic-phased array coil (Magnetom Skyra, Siemens Nederland B.V., The Hague, Netherlands). Imaging sequences included axial, coronal and sagittal T2-weighted images, as well as axial diffusion-weighted images. Experienced uroradiologists (>1000 scans) interpreted the scans using the PI-RADS v2 [14].

MRI was used as a stratification tool for prostate biopsy. According to hospital protocol, transperineal cognitive MRI-targeted biopsy was performed in patients with PI-RADS ≥ 4, with at least three cores per lesion and additional perilesional cores. In patients with PI-RADS 3, those with PSA density >0.15 (calculated using MRI-derived prostate volume) or DRE ≥T2 also underwent biopsy. Experienced uropathologists (>1000 biopsies) evaluated biopsy cores according to the International Society of Urological Pathology (ISUP) guidelines [15]. ISUP grade group (GG) ≥ 2 was considered csPCa; ISUP GG < 2 was considered cnsPCa. PI-RADS ≤ 3 were considered negative, whereas PI-RADS ≥ 4 was considered positive. For this analysis, PI-RADS 4–5 was used as the primary outcome, as these categories are generally considered an indication for prostate biopsy. Secondary outcomes included the potential reduction in hospital referrals and subsequent MRIs, and missed csPCa cases.

Data analyses were performed retrospectively, using RStudio (v4.4.0). Statistical significance was defined as *p* < 0.05. DRE data from GP referrals were used when available, supplemented by data recorded by the urologist when missing. Continuous variables were summarised as medians with interquartile ranges (IQR), and categorical variables as frequencies and percentages. Group differences were analysed descriptively to provide clinical context, using the Mann-Whitney U and the chi-square test.

Patients with DRE ≥ T2 were excluded from model development because of a direct indication for MRI according to current guidelines. Multivariable logistic regression analysis was conducted to evaluate the predictive value of IPSS, PSA, and age for PI-RADS≥4. Only variables accessible to GPs were included to ensure the model’s applicability in primary care. Age and PSA were analysed as continuous variables; IPSS was dichotomised according to the original grouping as ≤7 (no to mild LUTS) versus >7 (moderate to severe LUTS). Family history of PCa was excluded due to >50% missing data. We evaluated nonlinearity by restricted cubic splines. Model discrimination, defined as the ability to discriminate between PI-RADS < 4 or PI-RADS ≥ 4, was assessed using the receiver operating characteristic and the area under the curve (AUC) [16], [17].

Decision curve analysis was performed to evaluate the clinical utility of the model across a range of threshold probabilities. A clinically relevant threshold probability of 20% was defined a priori, corresponding to a trade-off where missing a positive MRI was considered approximately four times worse than performing an unnecessary MRI [18]. The net benefit of the model was compared with strategies of performing MRI in all patients (‘treat all’, reflecting current clinical practice) and in no patients (‘treat none’). Subsequently, to illustrate the impact of PSA and IPSS on predicted risk, we calculated estimated probabilities of a positive MRI for a series of hypothetical patients.

## 3 Results

A total of 409 patients were included in the study (Appendix 1), of whom 334 men without DRE ≥ T2 were included in the model development cohort (Table 1). Of those, 30% (101/334) had PI-RADS ≥ 4, of which 59% (60/101) had ISUP GG ≥ 2. Within the model development cohort, men with PI-RADS ≥ 4 were significantly older, had higher PSA and PSA density levels, and had a lower median prostate volume. Also, they more often reported no or few LUTS (IPSS ≤ 7).

**Table 1:** Patient characteristics of patients without DRE ≥T2

**Variables**	**Negative MRI (*n*** = **233)**	**Positive MRI (*n*** = **101)**	***p* value**
Age, median (IQR)	66 (61–71)	69 (62–73)	0.01 a
PSA at referral, median (IQR)	5.3 (4.4–6.6)	7.0 (5.1–10)	<0.001 a
PSA density, median (IQR)	0.1 (0.1–0.1)	0.2 (0.1–0.3)	<0.001 a
PSA density, *n* (%)			<0.001 b
<0.15 ng/ml	186 (79)	43 (43)	
Missing	0	1 (1.0)	
Positive family history*, n* (%)			0.96 b
Yes	32 (14)	15 (15)	
No	64 (28)	27 (27)	
Missing	137 (59)	59 (58)	
IPSS, median (IQR)	11 (7–15)	11 (5–17)	0.35 a
IPSS original grouping*, n* (%)			0.35 b
Mild	69 (30)	38 (38)	
Moderate	131 (56)	50 (50)	
Severe	33 (14)	13 (13)	
IPSS new grouping*, n* (%)			0.02 b
≤7	52 (22)	36 (36)	
>7	179 (77)	64 (63)	
Missing	2 (0.9)	1 (1.0)	
PIRADS*, n* (%)			
1	115 (49)	–	
2	90 (39)	–	
3	28 (12)	–	
4	–	66 (65)	
5	–	35 (35)	
MRI prostate volume, median (IQR)	55 (40–69)	40 (30–55)	<0.001 a

DRE = Digital Rectal Examination; IPSS = International Prostate Symptom Score; IQR = interquartile range; MRI = magnetic resonance imaging; PIRADS = Prostate Imaging Reporting and Data System; PSA = prostate-specific antigen.

a Mann-Whitney *U* test.

b Chi-square test.

The total IPSS could not be calculated for four patients due to incomplete questionnaires; these patients were excluded from the risk analyses. Multivariable logistic regression analysis was performed in 330 men without DRE ≥ T2 and showed statistical significance for PSA and IPSS (Table 2). Age was not found to be significant. Nonlinear modelling did not improve model fit for age or PSA (*p* > 0.05). The model showed fair discrimination, with an AUC of 0.68 (0.62–0.74).

**Table 2:** Multivariable logistic regression analysis among patients without DRE≥T2.

	**OR (95% CI)**	***p* value**
Age	1.04 (1–1.08)	0.05
PSA	1.17 (1.10–1.27)	<0.001
IPSS		
<7	Reference	0.04
≥7	0.57 (0.33–0.96)	
AUC (95% CI)	0.68 (0.62–0.74)	

AUC = area under the curve; CI = confidence interval; IPSS = International Prostate Symptom Score; OR = odds ratio; PSA = prostate-specific antigen; RR = relative risk.

**Fig. 1:**
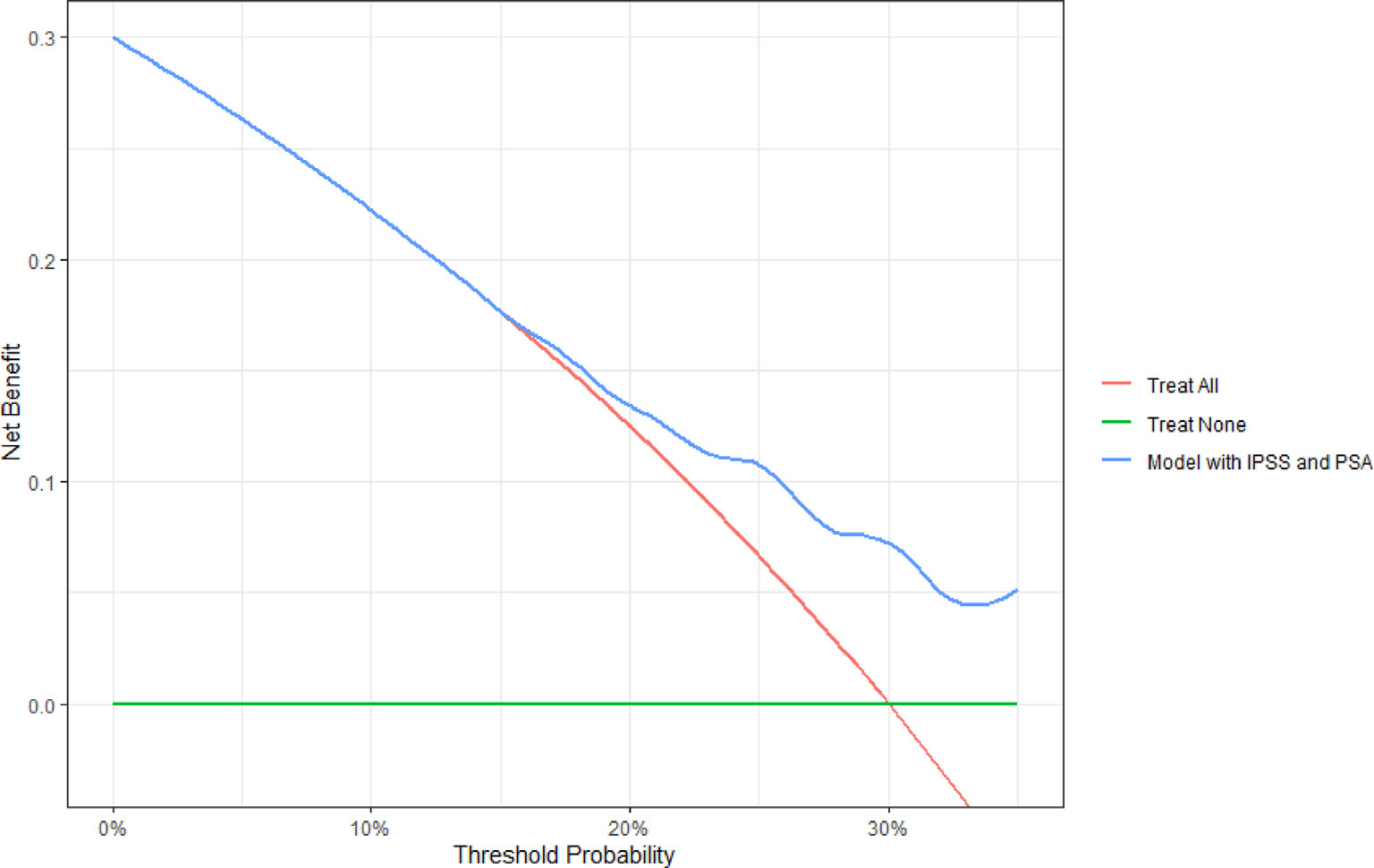
Decision curve analysis of the model incorporating PSA and IPSS for predicting a positive magnetic resonance imaging (MRI). The net benefit of the model (blue) is shown across a range of threshold probabilities and compared with default strategies of performing MRI in all patients (“treat all,” red) and in no patients (“treat none,” green). The model demonstrates a higher net benefit than the “treat all” strategy within an intermediate threshold range (approximately 17–30%) and consistently outperforms the “treat none” strategy across all thresholds. IPSS = International Prostate Symptom Score; PSA = prostate-specific antigen.

Decision curve analysis (Fig. 1) showed that the model incorporating PSA and IPSS yielded a higher net benefit than the “treat none” strategy across all threshold probabilities. Compared with the “treat all” strategy, the model demonstrated a higher net benefit within the threshold range of approximately 17–30%. At the predefined threshold probability of 20%, 254/330 MRIs (77%) would be performed, while 76/330 MRIs (23%) could be avoided. Based on the model-based referral decision, this resulted in 86/330 true positives (patients correctly referred with a positive MRI; 26%), 168/330 false positives (patients referred with a negative MRI; 51%), 63/330 true negatives (patients correctly not referred with a negative MRI; 19%), and 13/330 false negatives (patients not referred despite having a positive MRI; 3.9%). At this threshold, 3.9% of all patients would not be referred despite having a positive MRI, corresponding to 13/99 positive MRI findings (13%), including eight cases of missed csPCa. Table 3 presents threshold probabilities and corresponding numbers of performed and avoided MRIs.

**Table 3:** Diagnostic performance and clinical consequences of the prediction model across different threshold probabilities derived from the decision curve analysis with the model incorporating PSA and IPSS. For each threshold, patients with a predicted probability above the threshold would be referred for MRI, whereas those below the threshold would not. True positives represent patients correctly referred who were found to have a positive MRI (PIRADS ≥4), and false positives represent those referred who were found to have a negative MRI. True negatives represent patients correctly not referred with a negative MRI, whereas false negatives represent patients not referred despite having a positive MRI.

**Threshold**	**MRIs performed**	**MRI reduction**	**True positives**	**False positives**	**True negatives**	**False negatives**	**Missed csPCa cases among false negatives (total csPCa: 60/330)**
**17.5%**	305 (92%)	25 (7.6%)	96 (29%)	209 (63%)	22 (6.7%)	3 (0.9%)	3
**20%**	254 (77%)	76 (23%)	86 (26%)	168 (51%)	63 (19%)	13 (3.9%)	8
**22.5%**	218 (66%)	112 (34%)	79 (24%)	139 (42%)	92 (28%)	20 (6.1%)	9
**25%**	183 (56%)	147 (45%)	72 (22%)	111 (34%)	120 (36%)	27 (8.2%)	15

Among the hypothetical patients (Appendix 2), predicted probabilities of a positive MRI varied according to different combinations of PSA and IPSS, with lower IPSS scores (≤7) consistently associated with higher probabilities at comparable PSA levels. For example, at a PSA level of 3 ng/ml, a patient with IPSS > 7 had a lower predicted probability of a positive MRI (15%) compared with a patient with IPSS ≤ 7 (24%). At the predefined threshold probability of 20%, this difference would translate into different referral decisions, with patients with IPSS > 7 falling below the threshold and those with IPSS ≤ 7 exceeding the threshold.

## 4 Discussion

This is the first study to incorporate LUTS (measured by IPSS), into a model predicting MRI outcomes in primary care, with the aim of reducing unnecessary hospital referrals and MRIs in biopsy-naïve patients with suspected PCa. At a predefined threshold probability of 20%, the model could potentially reduce MRI referrals by 23% while missing 13% of positive MRI findings (corresponding to 3.9% of all patients). Higher IPSS scores (>7) were associated with a lower probability of PI-RADS ≥ 4 compared with lower IPSS scores (≤7), indicating that symptom burden meaningfully modifies predicted risk beyond PSA alone.

Our results showed an inverse association between IPSS and PI-RADS score, indicating that men with more pronounced LUTS were less likely to have suspicious MRI findings. To explore this further, we examined IPSS in relation to prostate volume and PSA density. Men with higher IPSS had larger prostates (49 vs 43.5 ml, *p* = 0.01), while PSA density was comparable (0.11 vs 0.12 ng/ml^2^, *p* = 0.28). This supports evidence that prostate enlargement contributes to benign obstruction and progression of symptom severity [19]. The comparable PSA density across IPSS categories suggests that PSA increases proportionally with gland size, reflecting benign rather than malignant activity. Although PSA density remains a strong predictor of csPCa, its discriminative performance is greatest in smaller prostates [20]. Nevertheless, BPH and prostate cancer may coexist in the same individual. However, prostate volume measurements required to calculate PSA density are not available in GP practice.

Few studies have investigated LUTS severity as a predictor in diagnostic pathways for PCa. Roobol et al [13] developed a risk calculator incorporating age, family history, and IPSS. However, this tool was developed for use in a setting where PSA levels were not available and was based on a screening population. Subsequent steps of ERSPC calculators did not further include the IPSS. Martin et al [21] found that severe LUTS predicted PCa in a screening population, in contrast to our findings, where IPSS > 7 correlated with lower PI-RADS ≥ 4 risk [21]. This discrepancy may be explained by the lack of MRI risk stratification before biopsy or by the age differences: 2% of our cohort were aged 44–51 years versus 85% in Martin et al’s [21] cohort, where moderate to severe LUTS was only observed in older menKlik of tik om tekst in te voeren.. As the prevalence of both LUTS and PCa increases with age, this may have confounded the observed association. Conversely, Franlund et al [22] reported a 22% lower PCa risk in men with LUTS, which is lower than the risk reduction we observedKlik of tik om tekst in te voeren.. However, they assessed LUTS with a single question focusing on obstructive symptoms, whereas our study assessed both obstructive and irritative symptoms [22], [23].

To our knowledge, no previous studies have used LUTS severity to predict MRI outcomes in clinical cohorts, making direct comparisons difficult. However, it is important to consider our results in context of existing research on MRI risk prediction tools. Mannaerts et al evaluated the RPCRC in 200 men with suspected PCa and reported a 37% reduction in MRIs and biopsies, missing only 2% of csPCa cases [1]. The strength of the RPCRC lies in the inclusion of TRUS-estimated prostate volume, a strong predictor of PCa, which improves model performance when compared with models based on PSA and DRE alone; however, it is generally not available in a GP setting [13], [24]. Similarly, in our study, adding prostate volume to our model would have improved discrimination to an AUC of 0.74 (95% CI = 0.68–0.80), with all variables (age, IPSS, PSA density) remaining significant. This highlights that the inclusion of LUTS may also provide additional benefit in secondary care. However, the need for TRUS increases hospital visits, costs, and patient burden. To mitigate this, Roobol et al [24], [25] proposed a model including DRE-estimated prostate volume, which Morote et al later validated in a nomogram, reducing MRI use by 22% while missing 5% of cancers [26]. However, DRE-estimated prostate volume remains less accurate than TRUS-estimated volume and shows limited diagnostic performance when performed by GPs (sensitivity 51%, specificity 59%) [24], [27]. Therefore, as our prediction model was designed for implementation in primary care, reliance on a model incorporating a validated questionnaire provides a more consistent approach than DRE-estimated volume, reducing errors and improving generalisability.

While we aimed to reduce unnecessary MRI scans, recent studies advocate MRI as a screening tool for PCa to avoid biopsy [28], [29]. Evans et al [28] demonstrated that MRI with a PI-RADS ≥ 4 threshold outperformed PSA alone in detecting PCa in a screening population. Our model could be used as a pre-MRI stratification tool to improve the cost-effectiveness of PCa screening. However, our model requires validation and recalibration in external or screening populations, as differences in LUTS prevalence, MRI findings (11% vs 36% PI-RADS ≥ 4), and csPCa rates (26% vs 66%) between population-based and GP-based cohorts may affect diagnostic accuracy [28].

Our study reported higher rates of negative MRI results than previous studies, as we classified PI-RADS ≤ 3 as negative instead of the conventional PI-RADS ≤ 2 [2], [3], [6], [30], [31]. However, PI-RADS 3 was deliberately excluded as positive due to its low positive predictive value (15%) compared with PI-RADS 4 (39%) and 5 (72%). Also, the PI-RADS ≥ 4 threshold improves interobserver agreement [32], [33].

Our study has several limitations. First, eight patients with csPCa would not have been referred for MRI at the selected threshold. Although these cases may be detected through PSA follow-up in primary care, this was not evaluated and remains uncertain. Second, while patients were prospectively identified, data collection was retrospective, resulting in substantial missing data for certain variables, such as family history. This may have reduced the completeness of the model and introduced information bias. In addition, four patients were excluded from the multivariable model due to incomplete questionnaire data. However, given the limited number of excluded patients, the risk of selection bias is considered low. Third, baseline comparisons between groups were exploratory and included multiple statistical tests. These analyses were included for descriptive purposes only and should be interpreted with caution in the context of prediction model development. Fourth, as model performance is case mix-dependent, the discriminative ability observed in our cohort should be interpreted within this context. External validation in a multicentre, preferably international, cohort is required prior to broader clinical implementation. Fifth, DRE data at referral were often missing and replaced with data recorded by urologists. Moreover, DRE by urologists was often only performed at the time of biopsy, that is, after a positive MRI result, introducing bias. Awareness of imaging findings may lead clinicians to classify equivocal DREs as normal, whereas in primary care, such uncertainty more often results in an abnormal assessment. As a result, equivocal DRE findings may be underrepresented in our cohort. Sixth, the model was developed for biopsy-naïve patients referred by GPs, limiting its generalisability to populations with other characteristics. Sixth, we used bpMRI instead of multiparametric MRI [4]. However, bpMRI is supported by the PI-RADS Steering Committee in biopsy-naïve patients, as recent studies confirm comparable diagnostic accuracy [34], [35], [36], [37]. Additionally, experienced uroradiologists ensured consistent assessment of the index lesions [38]. The model should be recalibrated for use in mpMRI-based pathways. Finally, although the IPSS questionnaire has been validated, it remains subjective and may be prone to response bias. In addition, the IPSS may not be suitable for patients with low literacy, a limitation that could be addressed by using the Visual Prostate Symptom Score [39].

## 5 Conclusion

In conclusion, this study developed a prediction model incorporating LUTS to improve risk stratification for hospital referral and MRI in biopsy-naïve patients with suspected PCa in primary care. At a predefined threshold probability of 20%, incorporation of LUTS could reduce MRI referrals by 23%, while 3.9% of all patients would not be referred despite having a positive MRI (13% of positive MRI findings), including eight cases of csPCa. This approach uses an easily available parameter to offer an efficient, resource-optimising strategy that supports risk-based triage and improves decision-making. We encourage future studies to externally validate our prediction model, particularly in cohorts using mpMRI, to enhance its generalisability.

  ***Author contributions***: Leonor Jane Paulino Pereira had full access to all the data in the study and takes responsibility for the integrity of the data and the accuracy of the data analysis.

  *Study concept and design:* Paulino Pereira.

*Acquisition of data:* Paulino Pereira.

*Analysis and interpretation of data:* Paulino Pereira.

*Drafting of the manuscript:* Paulino Pereira.

*Critical revision of the manuscript for important intellectual content:* Paulino Pereira, van Melick, Sedelaar, Blanker, van Leeuwen, van den Bergh.

*Statistical analysis:* Paulino Pereira.

*Obtaining funding:* None.

*Administrative, technical, or material support:* Paulino Pereira.

*Supervision:* van den Bergh, van Melick, Sedelaar.

*Other:* None.

  ***Financial disclosure:*** Leonor Jane Paulino Pereira certifies that all conflicts of interest, including specific financial interests and relationships and affiliations relevant to the subject matter or materials discussed in the manuscript (eg, employment/affiliation, grants or funding, consultancies, honoraria, stock ownership or options, expert testimony, royalties, or patents filed, received, or pending), are the following: None.

  ***Funding/Support and role of the sponsor:*** No funding or other financial support was received.

## Appendix A Supplementary data

Supplementary Data 1 This supplementary material contains baseline characteristics of the study population (Appendix 1) and estimated probabilities of a positive MRI result for hypothetical patients across PSA levels and IPSS categories (Appendix 2).
