# Supplementary material for: Incorporating Lower Urinary Tract Symptoms to Predict the Risk of Positive Prostate Magnetic Resonance Imaging
Source: Eur Urol Open Sci. 2026 Jun 13;90:1–7. doi: 10.1016/j.euros.2026.05.014 (PMC13280134; doi:10.1016/j.euros.2026.05.014)
Supplement: Supplementary Data 1 — This supplementary material contains baseline characteristics of the study population (Appendix 1) and estimated probabilities of a positive MRI result for hypothetical patients across PSA levels and IPSS categories (Appendix 2). [file mmc1.docx]

**SUPPLEMENTARY MATERIALS**

**APPENDIX 1**

**Table.** Patient characteristics of all patients.

| **Variables** | **Negative MRI (n=260)** | **Positive MRI (n=149)** | **p-value** |
| --- | --- | --- | --- |
| Age, *median (IQR)* | 66 (61-71) | 69 (62-73) | 0.01^*^ |
| PSA at referral, *median (IQR)* | 5.2 (4.2-6.7) | 7.2 (5.4-11) | <0.001^*^ |
| PSA density, *median (IQR)* | 0.1 (0.1-0.1) | 0.2 (0.1-0.3) | <0.001^*^ |
| PSA density, *n (%)*  <0.15 ng/ml  Missing | 207 (80)  1 (0.4) | 56 (38)  2 (1.3) | <0.001^†^ |
| Positive family history*, n (%)*  Yes  No  Missing | 40 (15)  73 (28)  147 (57) | 23 (15)  43 (29)  83 (56) | 0.98^†^ |
| DRE*, n (%)*  <T2  ≥T2  Not performed  Missing | 163 (63)  27 (10)  42 (16)  28 (11) | 88 (59)  48 (32)  2 (1.3)  11 (7.4) | <0.001^†^ |
| IPSS, *median (IQR)* | 11 (7-16) | 10 (5-16) | 0.06^*^ |
| IPSS original grouping*, n (%)*  Mild  Moderate  Severe | 78 (30)  142 (55)  40 (15) | 62 (42)  66 (44)  21 (14) | 0.05^†^ |
| IPSS new grouping*, n (%)*  ≤7  >7  Missing | 60 (23)  198 (76)  2 (0.2) | 54 (36)  94 (63)  1 (0.7) | <0.001^†^ |
| PIRADS*, n (%)*  1  2  3  4  5 | 125 (48)  105 (40)  30 (12)  -  - | -  -  -  85 (57)  64 (43) |  |
| MRI prostate volume, *median (IQR)* | 53 (40-69) | 40 (30-53) | <0.001^*^ |

**DRE** - Digital Rectal Examination; **IPSS** - International Prostate Symptom Score; **IQR** - Interquartile Range; **MRI** - Magnetic Resonance Imaging; **PIRADS** - Prostate Imaging Reporting and Data System; **PSA** - Prostate-Specific Antigen.

* Mann–Whitney U test
 † Chi-squared test

APPENDIX 2

**Table.** Estimated risk of a positive MRI result for hypothetical patients across a range of PSA values (3-9 ng/mL) and IPSS categories (≤7 vs >7).

| **Patient** | **PSA (ng/mL)** | **IPSS category** | **Predicted probability of positive MRI (%)** |
| --- | --- | --- | --- |
| **1** | 3 | ≤7 | 24 |
| **2** | 3 | >7 | 15 |
| **3** | 5 | ≤7 | 30 |
| **4** | 5 | >7 | 20 |
| **5** | 7 | ≤7 | 37 |
| **6** | 7 | >7 | 26 |
| **7** | 9 | ≤7 | 45 |
| **8** | 9 | >7 | 33 |

**IPSS** - International Prostate Symptom Score; **MRI** - Magnetic Resonance Imaging; **PSA** - Prostate-Specific Antigen.
